# Biases of Happy Faces in Face Classification Processing of Depression in Chinese Patients

**DOI:** 10.1155/2020/7235734

**Published:** 2020-08-17

**Authors:** Yuying Tong, Gang Zhao, Jinbo Zhao, Nianxiang Xie, Dong Han, Bowen Yang, Qi Liu, Hailian Sun, Yanjie Yang

**Affiliations:** ^1^Department of Psychology, School of Education, Heilongjiang University, China; ^2^Department of Child Health Care, Maternity and Child Healthcare, Hospital of Nanshan District, Shenzhen, China; ^3^Department of Human Movement and Sport Science, Harbin Sport University, China; ^4^Public Health College of Harbin Medical University, Heilongjiang Province, China; ^5^The First Affiliated Hospital of Harbin Medical University, China

## Abstract

We explored the face classification processing mechanism in depressed patients, especially the biases of happy faces in face classification processing of depression. Thirty patients with the first episode of depression at the First Affiliated Hospital of Harbin Medical University were selected as the depression group, while healthy people matched for age, gender, and educational level were assigned to the control group. The Hamilton Depression Scale and Hamilton Anxiety Scale were used to select the subjects; then, we used the forced face classification paradigm to collect behavioral (response time and accuracy) and event-related potential (ERP) data of the subjects. The differences between the groups were estimated using a repeated measurement analysis of variance. The total response time of classified faces in the depression group was longer than that in the control group, the correct rate was lower, and the difference was statistically significant (*P* < 0.05). N170 component analysis demonstrated that the latency of the depression group was prolonged, and the difference was statistically significant (*P* < 0.05). When classifying happy faces, the depressed patients demonstrated a decrease in N170 amplitude and a prolongation of latency in some brain regions compared with the healthy individuals. The cognitive bias in depression may be due to prolonged processing of positive facial information and difficulty in producing positive emotional responses.

## 1. Introduction

Depression is the leading cause of ill health and disability worldwide, and >300 million individuals live with depression [1]. Between 10% and 15% of patients with depression are at risk for suicide [2]. According to a survey, the economic burden in China due to depression exceeds 60 billion yuan [2].

Scholars have explored the etiologic mechanism underlying depression from genetic, biochemical, and psychosocial aspects, but the specific mechanism remains unclear. Cognitive factors, especially cognitive biases (processing biases to negative stimuli), are one of the major reasons for the occurrence, continuation, and development of depressive symptoms [3]. The cognitive model of depression demonstrates that depressed individuals tend to attribute negative meanings to neutral stimuli and enhance the treatment of depressive stimuli. Subsequently, many studies have confirmed that depressive patients tend to choose negative information consistent with their negative schema because of the negative cognitive schema in their brain when processing external information [4]. Studies have demonstrated that depressive patients have significant impairment of facial expression recognition [5]. The literature has also demonstrated that depressed individuals tend to exaggerate negative stimuli [6, 7] and are more excited about sad faces [8]. Depressed patients are more likely to judge happy faces as neutral stimulation, neutral faces as negative stimulation, and indistinguishable blurred faces as negative stimulation. Healthy individuals are more likely to interpret neutral expression as positive expression [6, 7]. Many event-related potential (ERP) studies have demonstrated that depressive patients have a bias for emotional processing and have a higher score in evaluating the intensity of sad faces [9].

However, the evidence has been inconsistent. Some scholars have not found that patients with depression have a special sense of perception (emotional and facial expression judgment) deficiencies [10]. Several studies have demonstrated no difference in the processing of emotional stimuli in major depressive disorder [6, 9, 11]; others have reported differences in the processing of either sad or happy expressions or both [4, 12]. Scholars have also posited that the impairment of negative information processing in patients with depression is due to the decline in positive expression recognition ability and the enhancement of negative expression stimulus recognition ability, which is closely related to the severity of depressive symptoms. Some scholars believe that the negative preference of depression is due to the decrease in the response to positive stimuli [13, 14].

Notably, in normal subjects, happy facial advantage (HFA) exists in normal face classification processing [10, 15], but in depressed patients, obstacles have been observed in processing happy facial expressions. Depressed patients have difficulty distinguishing positive social stimuli (e.g., happy expressions) [6, 16, 17]. The subtle difference is that patients with depression have a lack of positive emotion recognition, especially happy face recognition disorder, which may lead to the aggravation of a negative state or produce negative social effects. The score of happy face evaluation in patients with depression is significantly decreased and is significantly lower than that in the healthy and depression groups [18]. The study also confirmed that evaluation of positive emotional stimuli is decreased in depressed patients, which made them feel inadequate when processing positive materials. Depressed patients cannot be induced by positive stimuli to emerge from the depressive state, and depressed patients have a negative face attention preference [19]. The “attention avoidance” of positive faces has also been described which aggravates the negative cognitive bias of depressed individuals and makes it difficult for them to get rid of the negative mood and reach the normal state [20].

In summary, the results of the research on face classification and processing of depressed patients are inconsistent because of the differences in research paradigms, research methods, severity, and age of depression. According to other studies on emotional processing of depression, the most often used methods of emotional processing of depression have been the face recognition task [21], point detection task [22], and visual search task [16]. In contrast, the task of face classification based on classification speed has been less involved, and most of those studies have focused on the recognition of sadness and depression in patients with depression. A comparison of the accuracy of happy faces was also conducted [23]. Whether there is an HFA effect in the facial expression classification of depressed patients remains unresolved. Whether the classification pattern of depressed patients is the same as that of healthy individuals has not been reported in the relevant literature.

### 1.1. Aims of the Study

The aim of the present study was to determine whether the HFA effect exists in first-episode, drug-naïve, depressed patients, especially the biases of happy face in face classification processing of depression. The purpose of this study was to analyze the classification and processing mechanism of depressed patients' faces and to provide a basis for the diagnosis and clinical prediction of depression.

## 2. Materials and Methods

### 2.1. Subjects

The subjects for the study included 30 depressed patients and 30 healthy individuals selected from the outpatient department at a hospital in Harbin, China. The subjects in the depression and control groups were matched for gender, age, and educational level; there were no significant differences between the groups (*P* > 0.05). All the subjects were right-handed with normal or corrected vision. The experimental plan complied with the Helsinki Declaration and was approved by the Ethics Committee. After understanding the purpose and content of the study, all subjects signed an informed consent form.

Subjects were diagnosed with a first episode of depression, as described in DSM-IV. The subjects were between 18 and 55 years of age and took no psychiatric drugs, and the familial genetic history was unremarkable. The control and depression groups were matched for age, gender, and education level. The following criteria were also fulfilled: no serious physical diseases, no neuropsychiatric diseases, and no history of taking antianxiety, depression, and psychiatric drugs.

### 2.2. Methods

#### 2.2.1. Research Tools

The General Situation Questionnaire collected the gender, age, marital status, and educational level of the subjects, as well as whether they had taken psychotropic drugs within 1 month and whether there were depressed patients in the family.

*(1) Hamilton Rating Scale for Depression (HRSD)*. The HRSD was compiled by Hamilton. The evaluation method of the scale is simple, and the standard is clear. Seventeen items were used in this study. The scale was comprised of seven factors: anxiety, somatization, cognitive impairment, weight, day and night change, sleep disorder, retardation, and despair. If the total score of depression was <7, the individual had no symptoms of depression. If the total score of depression was >17, the individual may have mild or moderate depression. If the total score was >24, the individual may have severe depression [24].

*(2) Hamilton Anxiety Rating Scale (HAMA)*. The HAMA is comprised of 14 items divided into two dimensions (physical and mental anxiety). The scale is scored by five grades of 0–4 points (total score > 29 points, possible severe anxiety; total score > 21 points, obvious anxiety; and total score > 14 points, anxiety). The general demarcation value was 14 points.

#### 2.2.2. Stimuli

**Figure 1:**
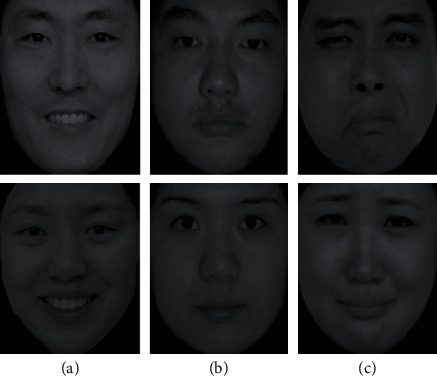
Example of the happy (a), neutral (b), and sad (c) faces used in the experiment.

The stimulus sequence was presented by E-prime software. The stimulus material was extracted from the facial system of the Chinese Emotional Picture System [25]. Ninety emotional faces, 30 happy faces, 30 neutral faces, and 30 sad faces were selected. The gender ratio of each face was 1 : 1. Examples of the stimuli are shown in Figure 1. The recognition and strong degrees between different facial expressions were obtained. No significant difference was observed in degree, and the size, brightness, contrast, and spatial frequency of all pictures were the same after software processing.

#### 2.2.3. Procedure

The experiment was conducted in a quiet and light-appropriate electrophysiology laboratory. The subjects sat in front of a 17-inch computer screen with a resolution of 1280 × 800 pixels. The refresh rate was 60 Hz. The distance between the eyes and the screen was 80 cm. The background of the experimental screen was light gray. The experiment was divided into two stages (practice and experiment stages). During the exercise, 18 pictures were presented. The subjects judged the facial expression information (happy, neutral, and sad) represented by the face, then responded to the key as soon as possible. The correct rate of practice reached >90%, and only then could the subjects enter the formal experiment.

In the formal experiment, there were four blocks. Each block contained 102 trials. Each face was presented for 300 ms. When the face was presented on the screen, the participants were asked to judge the facial expression information represented by the face (happy, neutral, and sad). Next, they were asked to press the button as soon as possible, to record the response time and correct rate of the participants. Each face was presented immediately and could not be the same. When three or more faces had the same expression, the keyboard adopted the principle of balanced matching. Before the experiment, 18 trials were trained. Between each block, participants could rest for 1–2 min.

#### 2.2.4. Electroencephalogram (EEG) Recording and Analysis

The EEG was recorded by 32 Ag/AgCl electrodes mounted on a custom-made cap (ECI; Eaton, OH, USA). According to the extended 10-20 system, the EEG was sampled with a 0.20–100 Hz band pass. The tip of the nose was used as a reference during recording, and electrode impedances were kept below 5 k *Ω*.

The EEG was segmented to obtain epochs starting 200 ms prior to and ending 800 ms following picture onset. Artifact correction for conventional artifacts (e.g., eye blinks) was performed by means of the “statistical correction of artifacts in dense array studies.” The EEG segments were averaged separately for each participant and for each face stimuli (at least 50 trials for each condition). The averaged waveforms were digital and had a low-pass of 30 Hz (24 dB/octave). Based on the literature [26, 27] and on scrutiny of the present scalp distribution of the N170 amplitudes, the statistical analysis was restricted to posterior lateral regions (T5, T6, A1, and A2) over the right hemisphere and the homolog sites over the left. For each subject, the peak of each ERP component [26, 27] was determined as the most negative peak between 120 and 220 ms (for N170). Subsequent visual scrutiny ensured that the most negative values represented real peaks rather than the endpoints of the epoch.

#### 2.2.5. Data Analysis

The general demographic data and behavioral results, such as age, education level, HRSD score, and HAMA score, were analyzed. The correct rate was calculated, and the wrong data were deleted. Next, the response time was analyzed by repeated measurement analysis of variance, with a *P* < 0.05 as the significant level of statistical difference.

These measurements were submitted to mixed model repeated measures three-way ANOVAs, with face emotion (happy, neutral, and sad), hemisphere (left and right), and site (A1/A2 and T5/T6) as the within-subject factors and group (depression and control) as the between-subject factor. Degrees of freedom were corrected whenever necessary using the Greenhouse–Geisser epsilon correction factor.

## 3. Results

### 3.1. Comparison of Demographic Data between the Depression and Control Groups

Table 1 presents the demographic data of the depression and control groups. There was no significant difference in the age and anxiety scores between the depression and healthy control groups (*P* > 0.05). The depression score of the depression group was higher than that of the obvious control group, and the difference was statistically significant (*P* < 0.05).

### 3.2. Behavioral Results

#### 3.2.1. Accuracy Comparison between the Depression and Control Groups

Types of facial expressions (happy, neutral, and sad) were used as within-group factors, and groups (depression and control groups) were used as between-group factors for repeated measurement of variance analysis. The results demonstrated that the main effect of the accuracy of the facial expression category was significant (*F*_(2, 57)_ = 28.81, *P* < 0.05, *η*^2^_*p*_ = 0.391). A significant difference was observed between the depression and control groups in the accuracy of facial expression classification (*P* < 0.05). The accuracy of classifying facial expressions in the depression group was significantly lower than that in the control group. The interaction between facial expressions and groups was nonsignificant (*F*_(2, 57)_ = 2.46, *P* > 0.05, *η*^2^_*p*_ = 0.061). The detailed information is shown in Table 2.

#### 3.2.2. Comparison of Reaction Time between the Depression and Control Groups

Before calculating the response time, the data collected from each participant were pretreated to delete the data beyond the standard deviation (±3). Approximately 7% of the data were deleted, and the classification response time of different facial expressions was deleted. The response time of the facial expression category between the depression and control groups is shown in Table 3. Using facial expression (happy, neutral, and sad) as the within-group factors and groups (depression and control groups) as the between-group factor, repeated measurement variance analysis demonstrated that the main effect of facial expression classification was significant (*F*_(2, 57)_ = 19.75, *P* < 0.01, *η*^2^_*p*_ = 0.405) and that the overall response time of the depression group (1203.35 ± 53.98 ms) was significantly longer than that of the control group (1036.89 ± 54.87 ms; Table 3); the difference was statistically significant (*P* < 0.05). No significant interaction was observed between facial expressions and groups (*F*_(2, 57)_ = 1.03, *P* > 0.05, *η*^2^_*p*_ = 0.034).

#### 3.2.3. Comparison of N170 Components between the Depression and Control Groups

**Figure 2:**
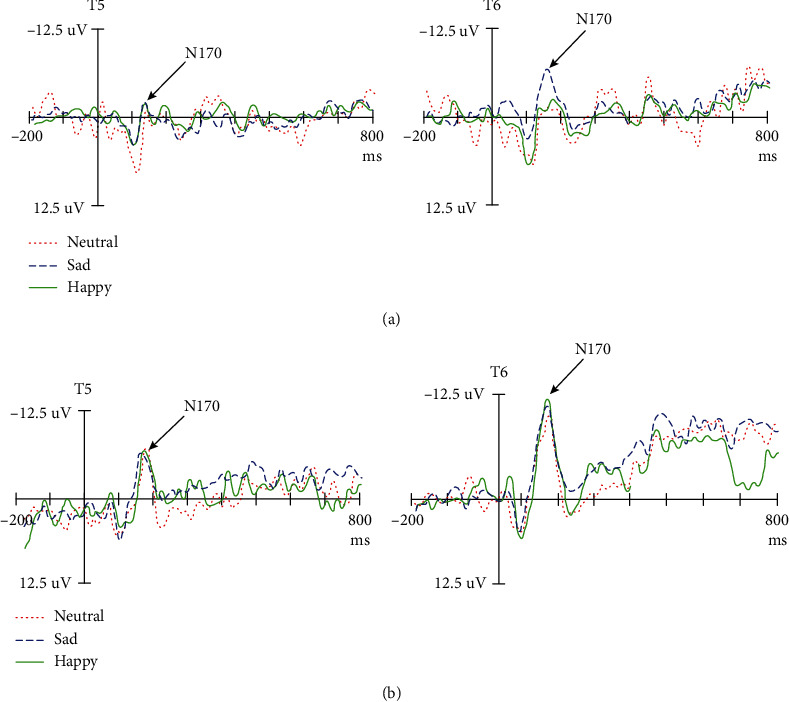
The wave of ERPs for facial expression category between the depressed (a) and control groups (b).

**Figure 3:**
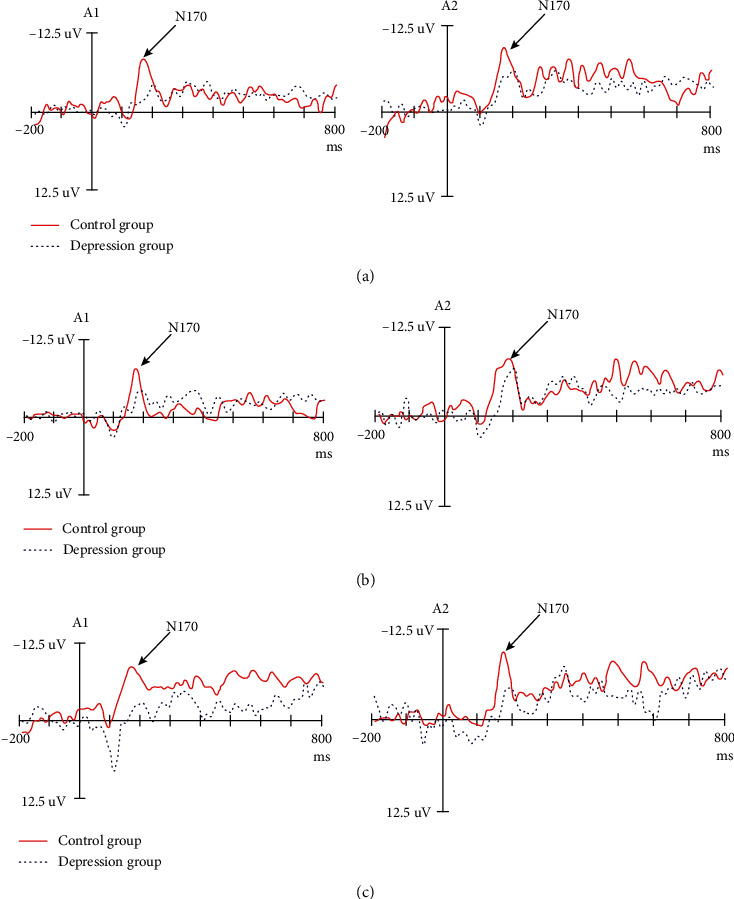
The amplitude and latency of facial expression ((a) happy face, (b) neutral face, and (c) sad face) between the depressed and control groups.

The results demonstrated that N170 was the most obvious negative component in the posterior temporal and occipital regions between 120 and 220 ms after the stimulus was presented (Figures 2 and 3).

The N170 amplitude of the T5 and T6 electrodes was analyzed. The results demonstrated no significant difference in the amplitude of N170 between the depression and control groups (*P* > 0.05). The results showed a significant difference in the main effect of facial expression (*F*_(2, 57)_ = 4.30, *P* < 0.05, *η*^2^_*p*_ = 0.137), the interaction effect of the facial expression × group (*F*_(2, 57)_ = 4.74, *P* < 0.05, *η*^2^_*p*_ = 0.149), and the interaction effect of the facial expression × group × hemisphere (*F*_(2, 57)_ = 3.44, *P* < 0.05, *η*^2^_*p*_ = 0.113). There was no significant difference in the interaction effects between the hemispheres (*P* > 0.05). Further analysis demonstrated that when classifying happy faces, there was a significant difference in the amplitude of N170 between the two groups (*P* < 0.05), whereas the amplitude of N170 in the depression group decreased (Figure 2). There was no significant difference in the amplitude of N170 between the depression and control groups when processing sad and neutral faces (*P* > 0.05). There was no significant difference in the amplitude of N170 of different facial expressions between the two groups (*F* < 1). In the control group, the amplitude of N170 induced by happy faces was the largest and significantly larger than that induced by neutral and sad faces (*F*_(2, 57)_ = 8.82, *P* < 0.05, *η*^2^_*p*_ = 0.246). There was no significant difference between the two groups (*P* > 0.05; Figure 3), and this finding indicates that the classification of facial expressions of depressive patients is impaired at this stage. There was no significant difference in the main hemispheric effect, hemisphere × group, hemisphere × facial expression, and hemisphere × facial expression × group (*P* > 0.05).

Analysis of N170 latency of the T5 and T6 electrodes demonstrated a significant difference between the two groups (*P* < 0.05), and the latency of the depression group was prolonged. There was no significant difference in the latency of N170 between the depression and control groups (*P* > 0.05; Figure 2) when classifying happy faces (*P* > 0.05). When classifying neutral and sad faces, the latency of the depression group was longer than that of the control group (*P* < 0.05).

The amplitude of N170 in A1 and A2 was analyzed. The results demonstrated no significant difference in the amplitude of N170 between the depression and control groups (*P* > 0.05). The main effect of facial expression was significant (*F*_(2, 57)_ = 5.35, *P* < 0.05, *η*^2^_*p*_ = 0.165). The interaction effect of the facial expression × group was significant (*F*_(2, 57)_ = 5.71, *P* < 0.05, *η*^2^_*p*_ = 0.175, and others). When classifying happy faces, there was a significant difference in the amplitude of N170 between the depression and control groups (*P* < 0.05), and the amplitude of N170 in the depression group decreased. For sad and neutral faces, there was no significant difference between the two groups (*P* > 0.05; Figure 3). There was a significant difference in the latency of different facial expressions between the depression and control groups (*F*_(2, 57)_ = 10.50, *P* < 0.05, *η*^2^_*p*_ = 0.280). Thus, the amplitude of N170 induced by happy faces in the control group was the smallest and significantly smaller than that induced by neutral and sad faces (Figure 3). There was no significant difference between the neutral and sad faces (*P* > 0.05), suggesting that the two groups had a different classification mechanism.

The latency of N170 in A1 and A2 was analyzed. The results demonstrated a significant difference in latency between the depression and control groups (*P* < 0.05). The main effect of facial expression was significant (*F*_(2, 57)_ = 3.54, *P* < 0.05, *η*^2^_*p*_ = 0.114). The latency of the depression group was prolonged. The interaction effect of the facial expression × group was significant (*P* < 0.05). When classifying different facial expressions, there was a significant difference in the N170 latency between the depression and control groups (*P* < 0.05). The classification latency of different facial expressions in the depression group was prolonged. There was no significant difference in the N170 latency of different facial expression classifications in depression groups (*F* < 1). In the control group, the latency of happy faces was significantly shorter than that of neutral and sad faces; there was no significant difference between the two groups (*P* > 0.05).

## 4. Discussion

### 4.1. Behavioral Analysis of Expression Classification Patterns in the Depression and Control Groups

In this study, the accuracy and response time of facial expression classifications were obtained using the task of facial expression classification. The overall response time of depressed patients was longer than that of the control group, and there was an HFA effect. These findings indicate that for depressive patients in the face classification processing stage, the reaction speed decreased, resulting in a slowdown in processing speed. At this stage, depressed patients may have cognitive processing disorders.

In this study, the face classification effect of different expressions in patients with depression was studied, and the results demonstrated that this was inconsistent with the literature [6, 18, 28]. The main reasons may be as follows. First, the stimulus materials used in this study were different from those used in previous studies. The stimulus materials used in this study were real faces of Chinese people rather than cartoon or distorted faces. Second, this study was based on the speed of the facial expression classification, and this is also one of the innovative strengths of this research. The most likely reason is that depressed patients have cognitive dysfunction in face classification.

### 4.2. Physiologic Mechanisms of Classification Patterns in the Depression and Control Groups

In this study, we were the first to analyze the electrophysiologic changes of facial expression classification in depressed patients compared to the facial expression classification patterns of the control group and attempted to explain the mechanism of cognitive impairment in depressed patients. We found no difference in the N170 amplitude between the depression and control groups in face classification processing, and the latency of N170 was prolonged. When classifying happy faces, the depression group demonstrated a decrease in N170 amplitude and a prolongation of latency in some brain regions compared with the control group. In contrast, when classifying sad and neutral faces, no significant difference was observed in the N170 amplitude between the depression and control groups.

Compared with the control group, the pattern of facial expression classification processing was different in the depression group, and there was no difference in the latency and amplitude of facial expression classification processing in the depression group. The results showed that there was no significant difference in the processing of facial expression in the patients with depression at the EEG level, and the HFA effect of depression disappeared. That is, the processing advantage of positive faces disappeared.

This study directly compared the individual level of faces (happy, neutral, and sad) and induces significant N170 components, which supports conclusions in the literature [29]. ERP studies have shown that the response of depressed patients to negative information has not increased, unlike the positive information that decreased with impaired processing [13, 30]. The cognitive impairment of depressed patients was only caused by the “absence” or “avoidance” of positive faces. This study was the first to elaborate on the positive “avoidance” of depressed patients from the perspective of face classification. We further confirmed that the positive avoidance of depression may originate from the classification processing stage before the early structural coding and the processing stage after the classification processing. At this stage, the cognitive control disorder of the positive stimulus in depressed patients leads to the relative gain of the negative stimulus processing relative to the positive stimulus and in the stimulus-oriented processing. In the context of work, depression has an early inhibitory effect on negative information [31], which suggests that depressed patients may have a higher perceptual sensitivity to negative emotional information. This negative biased brain electrophysiologic mechanism revealed that the occurrence of depression was partly due to the failure of the cognitive control system to regulate the production and evaluation of positive emotions, which provides a basis for further revising the negative biased theory of depression.

A notable phenomenon was found in this study. Our study focused on first-episode, drug-naive patients. The behavioral results showed that the interaction between facial expressions and groups was not significant and that the response time of depressed patients decreased. This finding is inconsistent with other studies [20]. At the EEG level, there was a significant difference in the interaction effect of the facial expression and groups with respect to latency and amplitude. The HFA effect of patients with depression disappeared. Specifically, the processing advantage of positive faces disappeared, and the depression group had no difference in classification processing of different facial expressions. There was no difference in the amplitude and latency of neutral and sad faces between the symptomatic and control groups. These results suggest a cognitive information processing bias in depressed patients. Notably, the deviation of expression classification information, the abnormal positive emotional response, and the decrease of ERP amplitude induced by happy faces in the depression group indicated that patients with depression paid less attention to happy faces and increased avoidance. In the classification of happy facial expressions in depressed patients, the ERP amplitude was significantly lower than the control group, and the latency was significantly longer than the control group. The first-episode, drug-naïve, depressed patients cannot fully perceive the positive material stimulus in classification stages, their evaluation of the positive stimulus is too low, and they cannot eliminate depression through the induction of the positive material, which aggravates the negative cognitive bias of depression and makes it difficult for patients with depression to escape the negative mood.

## 5. Limitations of This Study

The current study was limited by the number of cases in the experimental group. The experimental group was not large; however, all of the subjects were diagnosed with first-time depressive disorder by clinical psychiatrists. It is really difficult to collect a large number of patients in accordance with the diagnostic standard. As clinical samples, the number of cases in this study were not less than the majority of similar published studies. We will consider doing a follow-up corollary study in the future to verify our conclusions. Because of the low spatial resolution of ERP technology, it is necessary to coordinate with other cognitive research methods in many studies involving cognitive brain areas.

## Figures and Tables

**Table 1:** Comparison of demographic data between depression and control groups.

Variables	Depression group, *n* = 30	Control group, *n* = 30	*P* value
	Mean ± SD	Mean ± SD	
Age	44.88 ± 13.28	46.60 ± 9.41	0.217
Depression score	22.75 ± 3.10	3.38 ± 1.09	0.000
Anxiety score	4.37 ± 2.44	3.43 ± 1.14	0.063

**Table 2:** Accuracy (%) of facial expression category in the depression group and control group (*n* = 30).

Variables	Happy	Neutral	Sad
	Mean ± SD	Mean ± SD	Mean ± SD
Depression group	87.68 ± 7.50	82.87 ± 10.14	75.06 ± 13.32
Control group	95.97 ± 5.36	95.07 ± 5.48	78.77 ± 16.91

**Table 3:** Response time of facial expression category between depression and control groups (*n* = 30).

Variables	Happy	Neutral	Sad
	Mean ± SD	Mean ± SD	Mean ± SD
Depression group	1106.11 ± 356.10	1210.02 ± 327.03	1293.92 ± 301.74
Control group	857.12 ± 117.66	1115.25 ± 652.12	1138.31 ± 363.12

## Data Availability

The data used to support the findings of this study have not been made available because the data also form part of an ongoing study.
